# Health at the ballot box: disease threat does not predict attractiveness preference in British politicians

**DOI:** 10.1098/rsos.160049

**Published:** 2016-03-23

**Authors:** Gustav Nilsonne, Adam Renberg, Sandra Tamm, Mats Lekander

**Affiliations:** 1 Stress Research Institute, Stockholm University, Stockholm, Sweden; 2 Department of Clinical Neuroscience, Osher Center for Integrative Medicine, Karolinska Institutet, Stockholm, Sweden

**Keywords:** disease avoidance, behavioural immune system, disease threat, self-rated health, attractiveness, voting behaviour

## Abstract

According to disease avoidance theory, selective pressures have shaped adaptive behaviours to avoid people who might transmit infections. Such behavioural immune defence strategies may have social and societal consequences. Attractiveness is perceived as a heuristic cue of good health, and the relative importance of attractiveness is predicted to increase during high disease threat. Here, we investigated whether politicians' attractiveness is more important for electoral success when disease threat is high, in an effort to replicate earlier findings from the USA. We performed a cross-sectional study of 484 members of the House of Commons from England and Wales. Publicly available sexiness ratings (median 5883 ratings/politician) were regressed on measures of disease burden, operationalized as infant mortality, life expectancy and self-rated health. Infant mortality in parliamentary constituencies did not significantly predict sexiness of elected members of parliament (*p* = 0.08), nor did life expectancy (*p* = 0.06), nor self-rated health (*p* = 0.55). Subsample analyses failed to provide further support for the hypothesis. In conclusion, an attractive leader effect was not amplified by disease threat in the UK and these results did not replicate those of earlier studies from the USA concerning the relationship between attractiveness, disease threat and voting preference.

## 1. Introduction

As a part of human immune defence against infection, selective pressures have shaped adaptations that include behaviour. One set of such adaptations is reactive, that is, they increase the odds of survival through energy-saving motivational changes once infected [1]. Another set of adaptations is proactive and increases survival through disease threat detection by the triggering of disgust, anxiety and neophobia (fear of the new), leading to avoidance as the main propagated response [2]. When disease threat is high, tendencies for such behavioural patterns increase [2]. Watching pictures of people who display signs of disease [3], or pictures that evoke disgust [4,5], modulates inflammatory responses [3–5]. These and other findings suggest the existence of a disease avoidance system as one component of the immune system. Putatively because of the high cost of false-negative responses, humans appear to be sensitive to cues of disease, including a general set of superficial social cues that may signal increased risk for contagion [2].

Attractiveness is highly correlated to perceived health [6,7]. When disease threat is high, the relative importance of perceived signs of health in others may increase in importance. These phenomena have been extended to leadership, suggesting that disease threat may augment the preference for attractive leaders. White *et al*. reported that worse health predicted stronger preference for attractive electoral candidates, using a series of four experimental and observational studies [8]. In one of the experiments, attractiveness of an unreported number of major-party candidates from the 2010 US congressional elections was rated by 20 research assistants. Physical attractiveness was a significant predictor of electoral success, but only in areas with high disease threat, operationalized by a composite measure of infant mortality rate and life expectancy. In another experiment, White *et al*. used pictures of British politicians rated for sexiness on the website sexymp.co.uk. Participants who saw these pictures indicated that they were more likely to vote for sexier candidates, especially if disease concerns were experimentally activated.

Further support for disease avoidance theory in the context of elections is provided by a recent experimental study that tested whether health-related functional limitations affected the general preference for attractive leaders [9]. Using campaign portraits from US Senate races shown in pairs, participants rated attractiveness and were asked to choose the one for whom they would vote. Less healthy participants showed a stronger preference for more attractive contenders. This study was limited by its relatively small sample (*n* = 38).

We reasoned that if the prediction by White *et al*. holds, then ratings of attractiveness should be predicted by the disease threat in parliamentary constituencies. Higher disease threat would thus enhance the effect of physical attractiveness on the chance to be elected. This approach takes advantage of the large numbers of ratings on sexymp.co.uk, while using real-life voting behaviour as outcome.

In addition, we considered that self-rated health is a valid and relevant measure of disease threat. Self-rated health is an inclusive measure of global health that reflects comorbidity and with high predictive validity for morbidity, mortality, sickness absence and healthcare consumption [10,11]. Worse self-rated health is associated with more somatic symptoms and with higher levels of circulating pro-inflammatory cytokines [12–14]. Therefore, self-rated health is probably a more salient measure of disease threat than life expectancy or infant mortality, and we added it to infant mortality and life expectancy as predictors.

Thus, we aimed to investigate the relationship between local disease threat, including self-rated health as a predictor, and preference for attractive leaders across geographical regions.

## 2. Material and methods

On 7 February 2014, we obtained sexiness ratings for all members of the House of Commons on the sexymp.co.uk site. Politicians had been rated many times (median 5883, minimum 5184) in pair-wise comparisons. Sexiness (operationalized as fraction of wins) was regressed on disease burden data from parliamentary constituencies in England and Wales. Sex and political party were used as covariates of no interest. Infant mortality data for 2010–2012 were obtained from the United Kingdom Office for National Statistics (ONS) by ‘area of usual residence’ and averaged over the 3 years. To confirm that variation in infant mortality reflected genuine differences between constituencies, we performed simulations of infant mortality, showing that the observed variance (1.27) was greater than the variance expected from chance alone (0.47, for details, see the online materials [15]). Life expectancy data at birth were available by ‘local area’ for male and female newborns, respectively, and we calculated the average. The Pearson correlation between infant mortality and life expectancy was *r*(481) = −0.45 (95% CI −0.38, −0.52). This is lower than the correlation in the data used by White *et al*. (*r* = −0.78), and therefore infant mortality and life expectancy were analysed separately instead of using a compound measure. Areas were mapped to parliamentary constituencies by matching the areas either by unique codes (for life expectancy) or by name (for death rates) using the application programming interface (API) and data available at http://mapit.mysociety.org. Data for the health area that corresponded best with the constituency were used, with priority (i) health area and constituency were the same, (ii) the smallest area covering the constituency and (iii) the average of areas overlapping the constituency. The final number of observations for analysis was 484.

Self-rated health from the 2011 census was available from the ONS by parliamentary constituency for England and Wales. Health was rated on a 5-point scale, which we averaged for each constituency, higher values indicating better rated health. Analyses were performed using R v. 3.0.1 [16]. All code and data are available online [15].

## 3. Results

**Figure 1.**
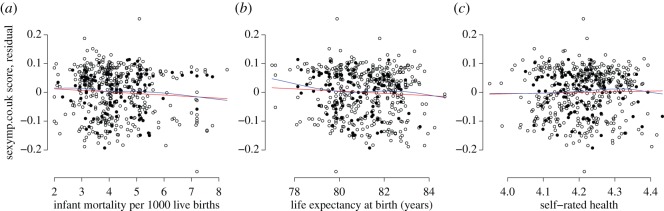
(*a*) Infant mortality and rated sexiness. (*b*) Life expectancy and rated sexiness. (*c*) Self-rated health and rated sexiness. Rated sexiness is shown as residuals after adjusting for political party and sex. Open circles are male members of parliament; closed circles are female members. Red lines show linear regressions; blue lines show loess predictions.

Infant mortality in the parliamentary constituency did not significantly predict sexiness of the elected member (figure 1*a*; *β* = −0.006, 95% CI = −0.013, 0.001, $r_{\mathrm{adj}}^{2}(475)=0.14,\;$ *p* = 0.08), nor did life expectancy (figure 1*b*; *β* = −0.006, 95% CI = −0.0131, 0.0002, $r_{\mathrm{adj}}^{2}(475)=0.14,\;$ *p* = 0.06), nor self-rated health (figure 1*c*, *β* = 0.030, 95% CI = 0.066, 0.125, $r_{\mathrm{adj}}^{2}(475)=0.14,\;$ *p* = 0.55).

Following the approach of White *et al.*, we also separately analysed the lowest-health constituencies, defined as more than 1 standard deviation from the mean, and found no significant relationship for infant mortality (*β* = 0.001, 95% CI = −0.032, 0.034, $r_{\mathrm{adj}}^{2}(51)=0.11,\;$ *p* = 0.94), life expectancy (*β* = −0.019, 95% CI = −0.049, 0.010, $r_{\mathrm{adj}}^{2}(71)=0.08,\;$ *p* = 0.20) nor self-rated health (*β* = −0.092, 95% CI = 0.634, 0.451, $r_{\mathrm{adj}}^{2}(80)=0.07,\;$ *p* = 0.74).

## 4. Discussion

This study investigated the relationship between disease threat and preference for attractive leaders by predicting sexiness in elected members of parliament from regional health indices. The effect of life expectancy on attractiveness preference was in the predicted direction, whereas the effect of infant mortality and self-rated health on attractiveness preference was in the opposite direction, while all effects were weak. Subsample analyses failed to provide further support for the hypothesis. Limitations of this study include self-selection of raters on the sexymp.co.uk website. The dataset used by White *et al*. included both winners and losers, whereas our dataset only included winners.

It is not clear why our results differ from those of White *et al*. [8]. It is possible that cross-cultural differences may explain some of the difference, or that the original effect size was overestimated (e.g. a Proteus phenomenon [17]). We thank Dr Steven L. Neuberg for suggesting during the review process the possible explanation that US candidates in the study by White *et al*. were less well known to their raters than the UK members of parliament, because the US participants did not rate politicians from their own electoral districts, and that politicians' physical appearance will be a less salient fitness cue if more is known about them. While this argument has strong validity on the face of it, the probability that the member of parliament from the rater's constituency would appear in any single comparison on sexymp.co.uk is low, as there are 650 constituencies. We therefore suspect that this effect is not a major explanatory factor.

In conclusion, the prediction by White *et al*. [8] that worse population health causes increased attractiveness preference did not apply in a different dataset using real voting behaviour. This suggests that further investigation is needed to clarify the role of disease threat in leadership choices, to investigate cross-cultural differences and to include areas with larger disease threat variance when compared with the USA and Britain.

## Data accessibility

Data and code are available at [15]: doi:10.5281/zenodo.12984.
